# Efficacy of alprostadil for preventing of contrast-induced nephropathy: A meta-analysis

**DOI:** 10.1038/s41598-017-01160-1

**Published:** 2017-04-21

**Authors:** Jing-Zhan Zhang, Xiao-Jing Kang, Ying Gao, Ying-Ying Zheng, Ting-Ting Wu, Long Li, Fen Liu, Yi-Ning Yang, Xiao-Mei Li, Yi-Tong Ma, Xiang Xie

**Affiliations:** 1 Department of Dermatology, People’s Hospital of Xinjiang Uygur Autonomous Region, Urumqi, P.R. China; 2 Department of Cadre ward, First Affiliated Hospital of Xinjiang Medical University, Urumqi, 830054 P.R. China; 3 Department of Cardiology, First Affiliated Hospital of Xinjiang Medical University, Urumqi, 830054 P.R. China

**Keywords:** Genetics, Genetic markers, Cardiovascular genetics

## Abstract

Contrast-induced nephropathy (CIN) has become the third-leading cause of hospital-acquired acute renal injury. Although alprostadil has been proposed as an effective preventative measure, this conclusion remains inconsistent. Thus, we performed a meta-analysis of the published studies on this topic to evaluate the preventative effect of alprostadil on CIN. Databases, including PubMed, the Web of Science, Cochrane Library, Wanfang, the China Biological Medicine Database (SinoMed) and the China National Knowledge Infrastructure (CNKI) were systematically searched. Nineteen clinical trials involving 2267 individuals were identified. We utilized a random or a fixed effect model to calculate the pooled odd ratios (ORs) and the standardized mean differences (SMD), respectively. Compared to the control group, the CIN risk decreased significantly in the alprostadil group (P < 0.00001, OR = 0.29, 95% CI = 0.21–0.39). In the subgroup of coronary angiography patients, the use of alprostadil also decreased the risk of CIN (P < 0.00001, OR = 0.27, 95% CI: 0.19–0.39). In conclusion, Alprostadil might be associated with a significant reduction in postcontrast Scr, BUN and CysC level and decrease the incidence of CIN.

## Introduction

With the development of radiography and the wide application of interventional therapy, the incidence of contrast-induced nephropathy (CIN) has become a serious clinical problem in the recent years. It has been turned into the third-leading cause of hospital-acquired acute renal injury^1, 2^. CIN is consistently defined as an increase in serum creatinine (Scr) levels of more than 25% or 44 μmol/L (0.5 mg/dL) of baseline levels, within 72 h after the procedure^3, 4^. However, the pathogenesis of CIN is not completely clear. It is believed that there is direct cellular toxicity of the contrast agent to renal tubules, resulting in the combined effects of flow dynamics, protein induced renal tubular obstruction and oxygen free radical damage^5, 6^. The main component of alprostadil is prostaglandin E1 (PGE1), which exists in the human body in the form of unsaturated fatty acid composition. It is a type of effective blood vessel-expanding agent, which can dilate kidney blood vessels, restrain platelet aggregation and thrombosis^7^. Alprostadil also decreases proteinuria and ameliorates renal function by increasing blood flow in renal and glomerular filtration rate (GFR)^8^. As early as 2000, Koch *et al*.^9^ studied 130 patients with pre-existing impaired renal function. Either placebo or a variable dose of PGE1 was administrated before contrast administration. The result revealed that postprocedure Scr levels were significantly lower in patients who received PGE1. Sketch *et al*.^10^ conducted a similar study in 2001 and found that using PGE1 (20 ng/kg/min) before radiocontrast exposure and its continued administration for a period of 5 to 5.5 hours can significantly reduce the level of Scr. However, there have been subsequently fewer related reports. In recent years, the effect of alprostadil for the prevention of CIN has attracted significant clinical and epidemiological research interest. Franz RW *et al*.^11^ designed a prospective, randomized, double blind study on 41 patients in 2011 and confirmed that oral PGE1 not only can reduce the incidence of CIN but also decrease Scr levels significantly. However, a systematic review and meta-analysis was still lacking. Thus, we performed a meta-analysis of the existing published data on this topic to evaluate the strength of the association.

## Results

### Literature search and study characteristics

**Figure 1:**
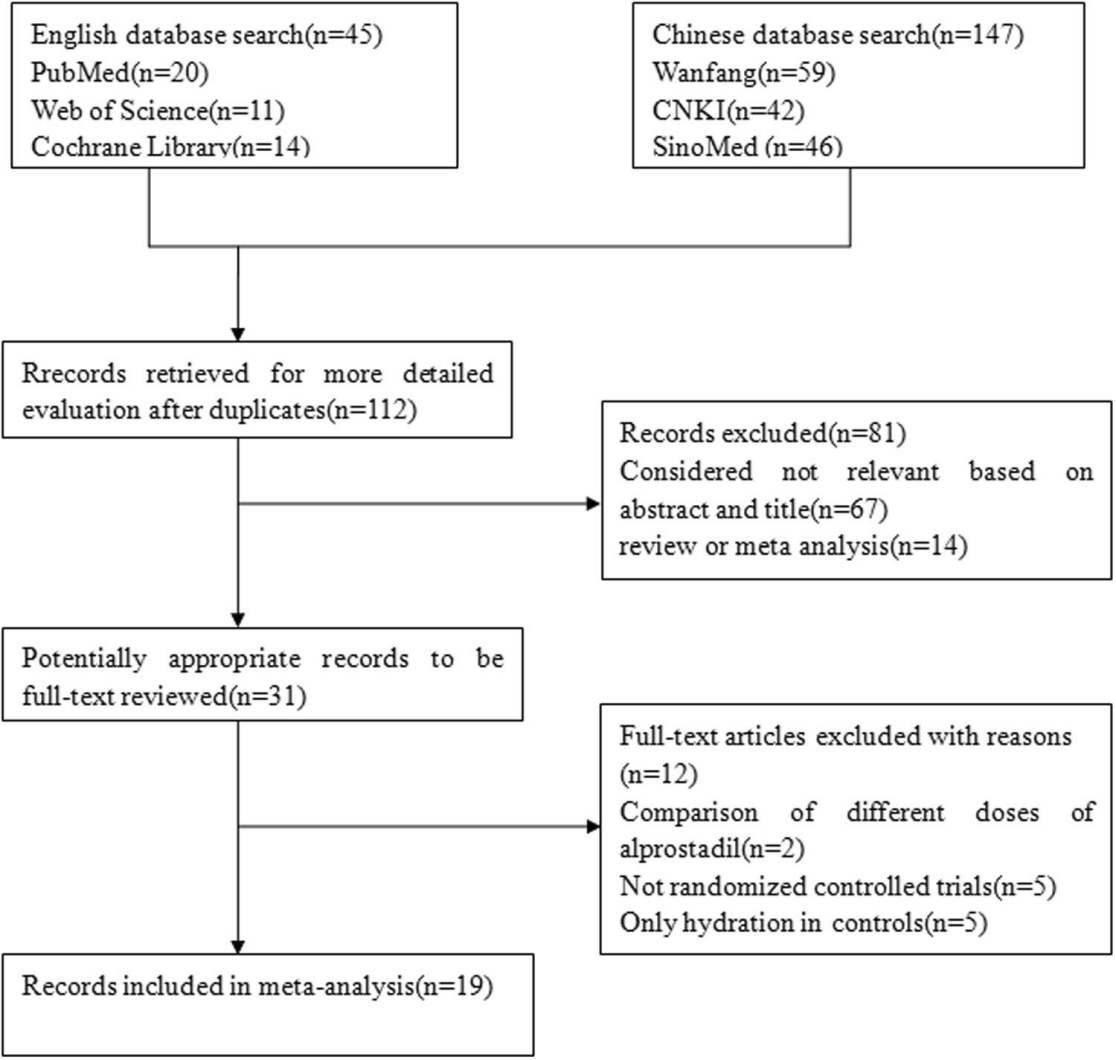
Flow diagram of study identification.

Briefly, we identified 192 studies from the original literature search. After duplicates and preliminary screening of the article titles and abstracts, 31 studies were considered of interest, these articles was retrieved for further evaluation. Thereafter, 12 out of 31 studies were excluded from the meta-analysis. Five studies were not RCTs^12–16^. In five studies, hydration was administered only in controls^17–21^. Two studies compared the different doses of alprostadil, and the data could not be extracted^22, 23^. Finally, a total of 19 RCTs^11, 24–41^ were included in the meta-analysis based on the above inclusion criteria. Of the 19 trials, 13 trials reported alprostadil to prevent CIN undergoing coronary angiography and/or intervention in coronary heart disease (CHD) patients. Thirteen studies^11, 24–30, 32–34, 36–40^ used hydration therapy in both cases and controls, but three studies did not^31, 35, 41^. In terms of contrast type, only 3 studies2^4, 27, 32^ used an iso-osmolar agent, others used a low-osmolar contrast agent. Figure 1 shows the process of literature retrieval. Table 1 shows the characteristics of the included studies. Regarding the quality of the included studies in our meta-analysis, the Jadad scores in the majority of the 19 studies were ≥2 points. The sample content in some studies was low.

**Table 1:** The characteristics of include studies.

First author	Publication year	Sample size		Age (years)		Object of study	NO. of CIN (case/control)	Observation index			Experimental group treatment strategy(alprostadil)	Jadad score
		Case(M/F)	Control(M/F)	Case	Control			BUN	CysC	Scr		
Franz R. W.^24^	2011	20 (12/8)	21 (17/4)	48–87	48–87	vascular surgery	0/1				oral 200 mg preoperative	4
Li B.^25^	2011	34 (34/0)	26 (26/0)	69.4 ± 7.3	67.9 ± 6.8	CHD CAG/PCI	2/3	48 h		48 h	20 μg + NS 20 ml iv qd, 30 min preoperative, continue 3d postoperative	2
Li J. Z.^26^	2016	50 (33/17)	53 (34/19)	58.11 ± 9.7	56.84 ± 9.29	CHD PCI	1/6			24 h, 72 h	10 μg + NS 100 ml iv drip qd, 1d preoperative, 3d postoperative	2
Liu H.^27^	2013	30 (16/14)	30 (15/15)	55–74	55–75	DM PAG	0/0		24 h, 48 h	24 h, 48 h	10 μg + NS 10 ml iv qd, 3d preoperative	3
Liu Y.^28^	2011	29 (16/13)	29 (19/10)	57.0 ± 7.5	59.4 ± 9.2	CHD CAG/PCI	4/13	48 h, 72 h		48 h, 72 h	10 μg + NS 50 ml iv pumping bid, 7d postoperative	2
Liu Y. Y.^29^	2011	82 (51/31)	84 (51/33)	60.8 ± 12.5	61.7 ± 12.8	CHD CAG/PCI	2/3			48 h	20ng/kg/min, continue 6 h postoperative	2
Li X. Y.^30^	2013	14 (8/6)	15 (11/4)	66 ± 12	66 ± 12	DM PAG or PTA	0/2		24 h, 72 h	24 h, 72 h	2ng/kg/min, continue 6 h preoperative and 20 μg + NS 40 ml iv qd, the second day, 5d postoperative	3
Li Y. N.^31^	2014	150 (96/54)	150 (54/96)	68.02 ± 7.03	68.49 ± 6.10	CHD CAG/PCI	4/13	72 h		72 h	10 μg + NS 100 ml iv drip qd, 0.5–1 h preoperative, 3d postoperative	3
Miao Y.^32^	2013	154 (120/34)	176 (133/43)	79.08 ± 6.16	78.26 ± 6.61	CECT	14/39		24 h, 48 h, 72 h	24 h, 48 h, 72 h	0.4 μg/kg/day, 48 h preoperative, continue to 48 h postoperative	4
Su C.^33^	2015	55 (35/20)	51 (33/18)	62.7 ± 10.8	63.5 ± 11.2	CHD PCI	2/7	72 h		72 h	10 μg + NS 100 ml iv drip qd, 1 day preoperative, 3d postoperative	2
Wang L.^34^	2016	50 (31/19)	50 (32/18)	60.48 ± 4.51	60.5 ± 4.17	CHD CAG/PCI	2/8	72 h		72 h	20 μg + NS 40 ml iv drip qd, 30 min preoperative, 0.5, 1, 2d postoperative	2
Wang Z. D.^35^	2015	65 (49/16)	63 (48/15)	58.2 ± 10.8	59.1 ± 11.2	CHD CAG/PCI	7/17	24 h, 48 h, 72 h		24 h, 48 h, 72 h	10 μg + NS 20 ml iv qd preoperative, 7d postoperative	3
Xu R.^36^	2012	30 (13/17)	30 (12/18)	60 ± 9	60 ± 11	CHD CAG/PCI	2/10			24 h, 48 h	10 μg + NS 20 ml iv qd, preoperative, 7d postoperative	2
Yan H. Y.^37^	2014	21 (12/9)	19 (11/8)	73.3 ± 6.3	75.1 ± 8.5	PAG	4/8	24 h, 48 h, 72 h	24 h, 48 h, 72 h	24 h, 48 h, 72 h	10 μg + NS 10 ml iv bid, 3d postoperative	2
Ye Y.^38^	2006	28 (25/3)	30 (26/4)	70.28 ± 5.6	72.62 ± 9.15	PAG/CECT	6/14	48 h, 72 h		48 h, 72 h	20 μg + NS 20 ml iv, qd, 3d postoperative	2
Zhao H. W.^39^	2014	58 (31/27)	58 (39/19)	64 ± 9	65 ± 8	CHD DM PCI	2/9		72 h	72 h	10 μg + NS 100 ml iv drip qd, 1d preoperative, 4d postoperative	2
Zhong S. G.^40^	2014	50 (38/12)	50 (39/11)	63.9 ± 7.6	64.1 ± 8.0	CHD DM PCI	1/8	72 h		72 h	100 μg + NS 100 ml iv drip bid, 3d postoperative	2
Zhou D. C.^41^	2013	112 (65)	103 (61)	62.1 ± 11.1	63.2 ± 10.9	CHD CAG/PCI	8/19				10 μg + NS 10 ml iv bid, 1d postoperative, 7–10d postoperative	3
Zhu L.^42^	2011	99 (99/0)	98 (98/0)	57 ± 15	57 ± 15	CHD CAG ± PCI	7/19	48 h	48 h	48 h	20 μg + NS 20 ml iv qd, 10d postoperative	2

CHD Coronary heart disease; DM diabetes mellitus; CAG coronary angiography; PCI percutaneous coronary intervention; PAG peripheral angiography; PTA percutaneous transluminal angioplasty; CECT contrast-enhanced computerised tomography;M male; F female; NS normal saline; d day; h hour; min minute; iv intravenous injection.

### Incidence of CIN

**Figure 2:**
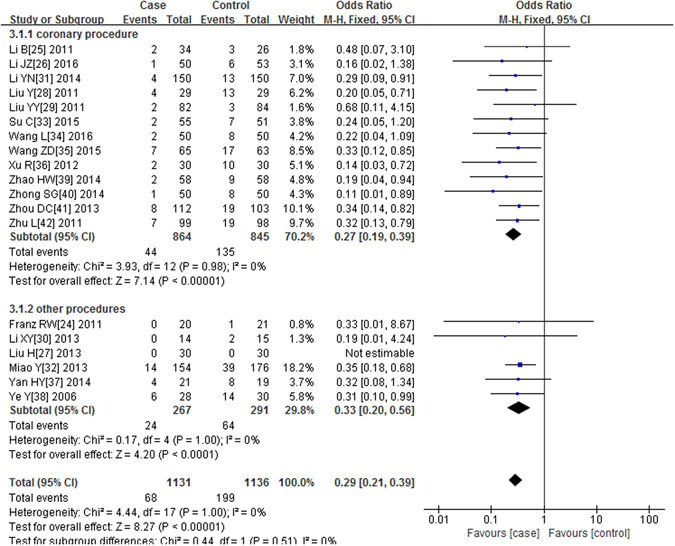
Forest plot of the association between using of alprostadil and the incidence of CIN, the horizontal lines correspond to the study-specific OR and 95% CI, respectively. The area of the squares reflects the study-specific weight. The diamond represents the pooled results of OR and 95% CI.

All of the 19 trials included in our meta-analysis reported the incidence of CIN. In the alprostadil group, 68 cases occurred in 1131 patients with CIN. There were 199 patients in the non-alprostadil group of 1136 patients with CIN. No significant heterogeneity was observed (P = 1.00, I^2^ = 0%), and the fixed effects model was applied to merge the ORs. The results suggested alprostadil administration significantly reduced the incidence of CIN (P < 0.00001, OR = 0.29, 95% CI = 0.21–0.39). To evaluate the effect of alprostadil in patients undergoing coronary procedure, we performed a subgroup analysis. In the subgroup of the coronary procedure, the use of alprostadil significantly reduced the risk of CIN (P < 0.00001, OR = 0.27, 95% CI = 0.19–0.39). In other procedures, the results are similar (P < 0.0001, OR = 0.33, 95% CI = 0.20–0.56), Fig. 2.

### Serum creatinine

**Figure 3:**
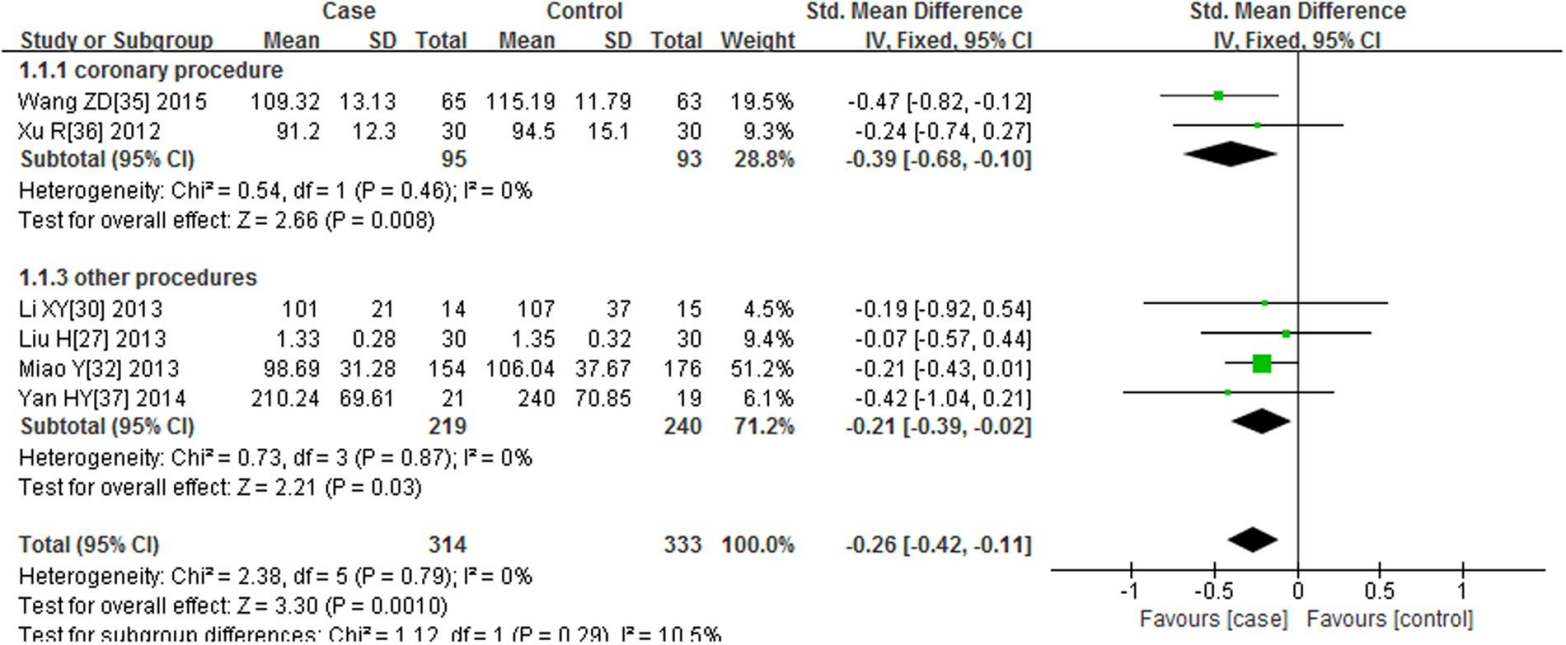
Forest plot of the association between using of alprostadil and the Scr level 24 hours after the contrast was administered, the horizontal lines correspond to the study-specific SMD and 95% CI, respectively. The area of the squares reflects the study-specific weight. The diamond represents the pooled results of SMD and 95% CI.

Six studies^26, 29, 31, 34–36^ had a measurement of Scr levels 24 hours after the contrast was administered. As shown in Fig. 3, no significant heterogeneity was found (P = 0.79, I^2^ = 0%), the fixed effects model was applied. The results showed that the postprocedural Scr levels were significantly decreased in the alprostadil group compared with the non-alprostadil group (P = 0.001, SMD = −0.26, 95% CI = −0.42, −0.11). In the subgroup analyses of the coronary procedure and other procedures, we obtained similar results (P = 0.008, SMD = −0.39, 95% CI = −0.68, −0.10 in the coronary procedure and P = 0.03, SMD = −0.21, 95% CI = −0.39, −0.02 in other procedures).

Ten articles^24, 26–28, 31, 34–37, 41^ had a measurement of Scr levels 48 hours after the contrast was administered. Heterogeneity was found among studies (P < 0.00001, I^2^ = 86%). The random-effects model was applied. The alprostadil group had a significantly lower postprocedural Scr level compared with the non-alprostadil group (P < 0.00001, SMD = −0.74, 95% CI = −1.08, −0.39). In the subgroup analyses of coronary procedure and other procedures, we obtained similar results (P = 0.001, SMD = −0.72, 95% CI = −1.15, −0.29 in the coronary procedure and P = 0.03, SMD = −0.79, 95% CI = −1.50, −0.07 for other procedures).

Twelve articles^25, 27, 29–34, 36–39^ had a measurement of Scr level 72 hours after the contrast was administered. Heterogeneity was observed among studies (P < 0.00001, I^2^ = 88%). The random-effects model was applied. The alprostadil group had a lower postprocedural Scr level compared with the control group after 72 h (P < 0.00001, SMD = −0.78, 95% CI = −1.10, −0.46). Similar results were obtained in the subgroup of the coronary procedure (P = 0.0002, SMD = −0.79, 95% CI = −1.20, −0.38) and other procedures (P = 0.03, SMD = −0.78, 95% CI = −1.48, −0.08). We presented the comparison of Scr level 24 h after procedure in the Fig. 3. Other data did not shown.

### Blood urea nitrogen

Two studies^34, 36^ had a measurement of the blood urea nitrogen (BUN) level 24 hours after the contrast administeration. No significant heterogeneity was observed (P = 0.42, I^2^ = 0%), and the fixed effects model was applied. The results showed that the postprocedural BUN level was significantly reduced in the alprostadil group compared with the non-alprostadil group (P = 0.004, SMD = −0.45, 95% CI = −0.76, −0.14). Only 2 studies were included, and we did not perform subgroup analysis.

**Figure 4:**
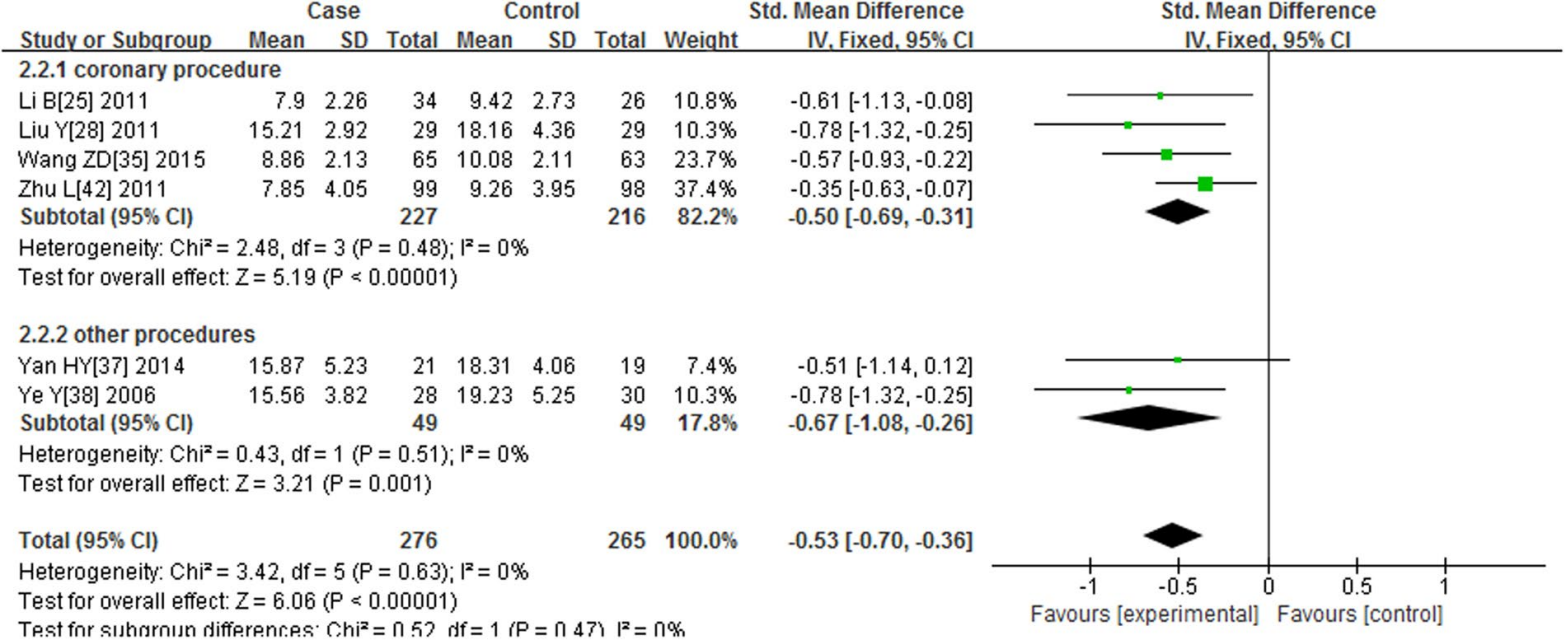
Forest plot of the association between using of alprostadil and the BUN level 48 hours after the contrast was administered, the horizontal lines correspond to the study-specific SMD and 95% CI, respectively. The area of the squares reflects the study-specific weight. The diamond represents the pooled results of SMD and 95% CI.

As shown in Fig. 4, six studies^24, 27, 34, 36, 37, 41^ had a measurement of BUN level 48 hours after the contrast was administered. No significant heterogeneity was observed (P = 0.63, I^2^ = 0%), and the fixed effects model was applied. The alprostadil group had a significantly lower postprocedural BUN level compared with the non-alprostadil group after 48 h (P < 0.00001, SMD = −0.53, 95% CI = −0.70, −0.36). In the subgroup analyses of coronary procedure and other procedures, the results were similar (P < 0.00001, SMD = −0.50, 95% CI = −0.69, −0.31 in the coronary procedure and P = 0.001, SMD = −0.67, 95% CI = −1.08, −0.26 for other procedures).

Eight studies^27, 30, 32–34, 36, 37, 39^ had a measurement of the BUN level 72 hours after the contrast was given. Heterogeneity was found after the 72 h (P < 0.00001, I^2^ = 90%). The random effects model was applied. The alprostadil group had a significantly lower postprocedural BUN level compared with the control group after 72 h (P = 0.0004, SMD = −0.84, 95% CI = −1.31, −0.38). Similar results were found in the subgroup of coronary procedure (P = 0.003, SMD = −0.86, 95% CI = −1.43, −0.28) and other procedures (P < 0.0001, SMD = −0.83, 95% CI = −1.25, −0.42).

### CysC

Four studies^26, 29, 31, 36^ had a measurement of the CysC level 24 hours after the contrast was administered. Heterogeneity was observed (P = 0.02, I^2^ = 77%), and the random effects model was applied. The results showed that the postprocedural CysC level significantly decreased in the alprostadil group compared with the non-alprostadil group (P = 0.02, SMD = −0.54, 95% CI = −1.01, −0.08). None of the 4 studies observed CHD patients undergoing a coronary procedure.

**Figure 5:**
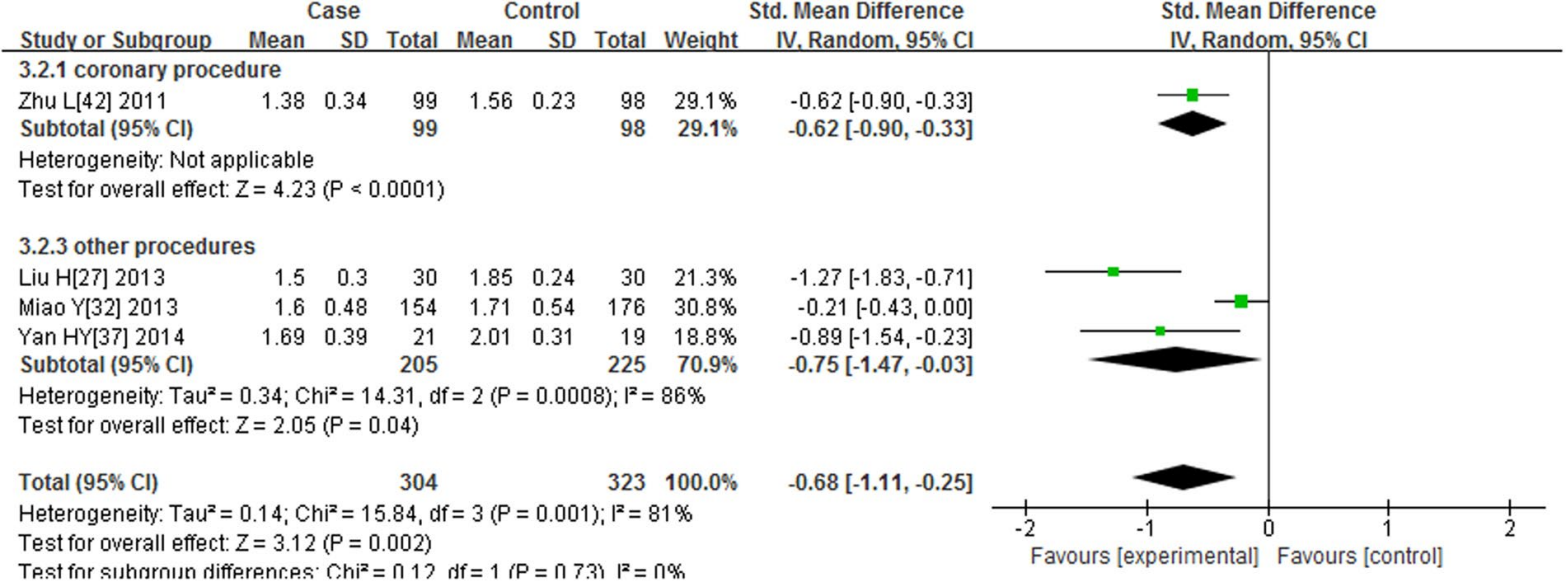
Forest plot of the association between using of alprostadil and the CysC level 48 hours after the contrast was administered, the horizontal lines correspond to the study-specific SMD and 95% CI, respectively. The area of the squares reflects the study-specific weight. The diamond represents the pooled results of SMD and 95% CI.

As shown in Fig. 5, four studies^26, 31, 36, 41^ had a measurement of the CysC level 48 hours after the contrast was administered. Heterogeneity was found (P = 0.001, I^2^ = 81%), and the random effects model was applied. The results showed that the postprocedural CysC levels were significantly reduced in the alprostadil group compared with the non-alprostadil group (P = 0.002, SMD = −0.68, 95% CI = −1.11, −0.25). Only 1 study observed CHD patients undergoing the coronary procedure (P < 0.0001, SMD = −0.62, 95% CI = −0.90, −0.33). Similar results were found in the subgroup of other procedures (P = 0.04, SMD = −0.75, 95% CI = −1.47, −0.03).

Four studies^29, 31, 36, 38^ measured the CysC level 72 hours after the contrast was administered. Heterogeneity was observed (P < 0.00001, I^2^ = 89%), and the random-effects model was applied. The alprostadil group had a significantly lower postprocedural CysC level compared with the control group (P = 0.63, SMD = 0.17, 95% CI = −0.51, 0.84). In the subgroup of coronary procedure, we found similar result (P = 0.57, SMD = 0.37, 95% CI = −0.90, 1.64). There was one study^36^ focused on elderly patients with renal insufficiency that provided the level of SysC 72 h after the contrast was administered, but the result of this trial was not combined with the findings of other studies.

### Sensitivity analysis

The contribution of each included study to the pooled estimate was performed to assess the sensitivity analysis. The sensitivity analysis of the incidence of CIN was limited to the 19 published trials. We excluded individual studies one at a time and recalculated the pooled P or OR estimates for the remaining studies. No studies had an undue influence on the pooled P or OR estimates. Moreover, their data did not substantially change the pooled point estimate when the fixed effects model transformed into random-effects model in all studies. Six studies had reported the postprocedural Scr level in angiography after 24 hours. In the study of the BUN level 24 hours after the contrast was administered, only two studies were included, Wang *et al*.^34^ had an undue influence on the pooled P or SMD estimates. There were 4 studies reporting the postprocedural CysC level 24 hours after the contrast was administered, and Liu *et al*.^26^ and Yan *et al*.^36^ had an undue influence on the pooled P or SMD estimates. However, their data did not substantially change the pooled point estimate when converting from the random-effects model to the fixed effects model in all studies. Thus, our results are relatively reliable.

### Publication Bias

**Figure 6:**
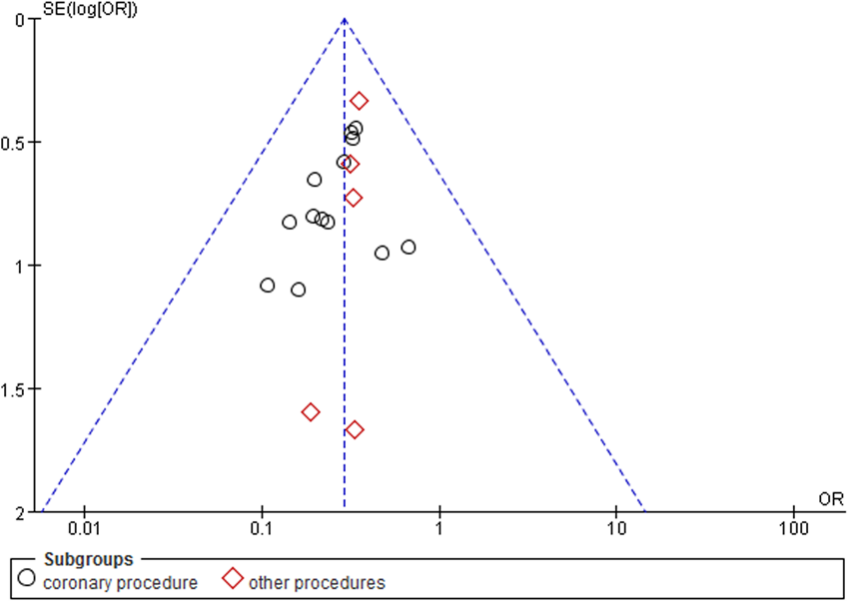
Funnel plot for publication bias test between using of alprostadil and the incidence of CIN. Each point represents a separate study for the indicated association. The horizontal and vertical axis correspond to the OR and confidence limits. OR odds ratio, SE standard error.

The publication bias of each study was evaluated using funnel plots, and the publication bias was found to be low in the current meta-analysis. In the CIN studies, no visual publication bias was found in the funnel plot (Fig. 6). Besides, no visual publication bias was found in the funnel plot in the studies on the postprocedural Scr, BUN, and CysC levels.

## Discussion

With the development of interventional and modern image techniques, the application of contrast agent is even more extensive. The subsequent CIN is increasing progressively, which has become one of the important reasons for acute renal failure. This finding is second only to renal insufficiency and renal toxicity drugs. Among all of the interventional treatments, the CIN of PCI was the highest, at 3% to 2%^42^. This is not easy to detect early, since the majority of patients have no obvious clinical symptoms for CIN except for some patients with renal failure. After CIN, the patient was hospitalized for a long time, and the short-term mortality and medical burden also increased^43^. There is no effective treatment for CIN, and thus, the prevention of CIN has become the primary task in the clinic.

Drug prevention, including the use of statin, N-acetylcysteine, hydration with normal saline or sodium bicarbonate, and iso-osmolar contrast medium have been proposed to prevent the development of CIN^44–48^. Spargias *et al*.^49^ performed a randomized, double-blind and placebo-controlled trial of iloprost in 208 patients and found that iloprost may protect against CIN in high-risk patients undergoing a coronary procedure. Ye *et al*.^50^ published a meta-analysis about the effect of alprostadil on preventing contrast-induced nephropathy for PCI in diabetic patients, but they did not research about the general population and patients with non PCI. It still lacks consistent and robust evidence that alprostadil decreases the incidence of CIN and improves renal function. Thus, we performed this meta-analysis.

Our meta-analysis suggests that alprostadil might be associated with a significant reduction in the postcontrast Scr, BUN and CysC level and decrease the incidence of CIN. In the subgroup of patients undergoing coronary angiography with or without PCI, we obtained similar results. These results are based on small sample size studies and require further exploring.

Heterogeneity was found in our meta-analysis of postcontrast Scr, BUN and CysC levels. It is a potential problem that may affect the interpretation of these results. We performed a subgroup analysis by following the type of contrast agent operation and divided these operations into two subgroups: patients undergoing coronary angiography with or without PCI and patients undergoing other procedures. The heterogeneity in the subgroups was still relatively large. Heterogeneity may be attributed to potential confounding factors, resulting from diversity in sample-sizes, age, usage and dosage of alprostadil, contrast dose and types, hydration method, chronic kidney disease, experimental methods, and other factors.

To better interpret the results, other limitations of our meta-analysis should also be recognized. First, the relative paucity of quality data and some inevitable publication bias may exist in our results. Only the full text studies in Chinese and English were included in this meta-analysis. Thus, some unpublished or reported studies in other languages may not be incorporated. Cultural background factors can also affect the decision to publish, making researchers more or less likely to report or edit negative results in some areas of research. Second, the included studies were of short duration, and only small trials to measure such patient-centered outcomes, such as the need for renal replacement therapy, the length of hospital stay and the in-hospital mortality rate. We did not have access to sufficient data to determine whether preexisting decreased kidney function and other risk factors (e.g., usage and dosage of Alprostadil, contrast dose and types, hydration method and age) could influence the effect of alprostadil on the risk of contrast-induced nephropathy. Some other factors, such as HDL cholesterol level may also be associated with CIN^51^, and we do not have further study. Furthermore, most of the trials included in the meta-analysis consisted of patients undergoing coronary angiography with or without PCI. The other types of procedures were incorporated less, for example, peripheral angiography, percutaneous transluminal angioplasty and contrast-enhanced computerized tomography. This may affect the representativeness of our meta-analysis. Lastly, our meta-analysis only had a measurement of the Scr, BUN and CysC level 24, 48, and 72 h after the contrast was given, and more RCTs may be needed to determine the long-term efficacy of alprostadil treatment for the improvement of renal function. Despite these limitations or disadvantages, our meta-analysis had some advantages. This is the first meta-analysis that consolidates the available information to date regarding the use of alprostadil in the prevention of CIN. A systematic review of the association of alprostadil and the incidence of CIN may overcome the limitation of the small sample sizes of the study populations by increasing the sample size, thereby generating more robust data. Moreover, the quality of the case-controlled studies included in our meta-analysis was satisfactory and met our inclusion criteria.

## Conclusion

Our meta-analysis suggests that alprostadil might be associated with a significant reduction in the postcontrast Scr, BUN and CysC level and maybe decrease the incidence of CIN. In the subgroup of patients undergoing coronary angiography with or without percutaneous intervention, we obtained similar results. These results were based on random clinical trial studies and require further verification.

## Methods

### Search strategy

Databases, including PubMed, Web of Science, Wanfang Data, the China Biological Medicine Database (SinoMed) and the China National Knowledge Infrastructure (CNKI) were systematically searched. Only English and Chinese language articles published before December 2016 were included. Reviews and editorials are excluded. The following keywords were used for the searching: “Alprostadil” OR “Prostaglandin E1” OR “PGE1” AND “contrast-induced nephropathy” OR “CIN” OR “Renal insufficiency” OR “Acute renal injury”. The reference lists of the included articles s that met our inclusion criteria were also searched in order to find potentially relevant titles. A study of the reference list, in line with our inclusion criteria was also searched for potentially relevant titles.

### Selection criteria

We included studies that met the following criteria: (1) clinical trials of human adults without ethnic restriction; (2) patients undergoing a contrast-using procedure, regardless of the type of procedure; patient comparisons between the alprostadil group and the control group, and foundation treatment is the same in the two group; (3) the primary outcome is CIN incidence, and the definition of CIN is clearly presented in every study. (4) independent of the potentially relevant results in kidney function before and after using contrast media, the serum creatinine level (Scr), blood urea nitrogen (BUN) and cystatin C (CysC) is also assessed and included. The baselines before exposure to contrast media were similar; (5) is a randomized controlled trial (RCT). Studies were excluded from analysis when (1) it was not possible to extract data from the published results, and (2) they contained republished data.

### Data Extraction

Two authors (JZ Zhang and TT Wu) independently extracted data from the included studies. Disagreements were resolved by consensus. If these two authors can not reach a consensus, then the result was reviewed by the third author (X Xie). The extracted data consisted of the follow items: the first author’s name, publication year, sample size, and age (in years), object of study, number of CIN, observation index, treatment strategy, and Jadad score.

### Quality assessment

To determine the methodological quality of every study, RCTs were evaluated using the Jadad quality scale, which rates aspects of randomization, blinding, and withdrawals^52^. A score of 3 or higher was considered good quality. Two investigators (Y Gao and L Li) independently assessed the quality of the included studies. Then, the results were reviewed by a third investigator (YT Ma). Disagreement was resolved by discussion.

### Statistical analysis

We compared the CIN incidence and the postcontrast Scr, BUN, CysC levels between the alprostadil groups and control groups to determine if they are significantly different. Dichotomous data (CIN incidence) were analyzed using odds ratios (ORs) and 95% confidence interval (CI), whereas continuous variables (Scr, BUN, CysC), which was expressed as the mean ± SD were analyzed using the standardized mean differences (SMD) and 95% CI. Heterogeneity between included studies was assessed by I^2^ statistic, and P < 0.10 and I^2^ > 50% indicated evidence of heterogeneity^53, 54^. If heterogeneity existed among the studies, then the random effects model was used^55^. Otherwise, the fixed effects model was adopted^56^. Sensitivity analyses were performed to assess the heterogeneity and robustness of the pooled results. The potential publication bias was investigated using the funnel plot. Analyses were performed using RevMan 5.33 (Cochrane Collaboration, The Nordic Cochrane Centre, Copenhagen).
