# Author Correction: Efficacy of alprostadil for preventing of contrast-induced nephropathy: A meta-analysis

**DOI:** 10.1038/s41598-019-39023-6

**Published:** 2019-05-07

**Authors:** Jing-Zhan Zhang, Xiao-Jing Kang, Ying Gao, Ying-Ying Zheng, Ting-Ting Wu, Long Li, Fen Liu, Yi-Ning Yang, Xiao-Mei Li, Yi-Tong Ma, Xiang Xie

**Affiliations:** 1 Department of Dermatology, People’s Hospital of Xinjiang Uygur Autonomous Region, Urumqi, P.R. China; 2 Department of Cadre ward, First Affiliated Hospital of Xinjiang Medical University, Urumqi, 830054 P.R. China; 3 Department of Cardiology, First Affiliated Hospital of Xinjiang Medical University, Urumqi, 830054 P.R. China

Correction to: *Scientific Reports* 10.1038/s41598-017-01160-1, published online 21 April 2017

This Article contains errors in the first column of Table 1, where the reference numbers are incorrect. All these articles are already referenced in the Article, and the Table with the corrected reference numbers is included below.

In addition, Table 1 contains an error in the ‘Experimental group treatment strategy(alprostadil)’ for the study by Franz RW [11] where,

“oral 200 mg preoperative”

should read:

“oral 200 μg preoperative”

**Table 1:** The characteristics of included studies. CHD Coronary heart disease; DM diabetes mellitus; CAG coronary angiography; PCI percutaneous coronary intervention; PAG peripheral angiography; PTA percutaneous transluminal angioplasty; CECT contrast-enhanced computerised tomography; M male; F female; NS normal saline; d day; h hour; min minute; iv intravenous injection.

First author	Publication year	Sample size		Age (years)		Object of study	NO. of CIN (case/control)	Observation index			Experimental group treatment strategy(alprostadil)	Jadad score
		Case(M/F)	Control(M/F)	Case	Control			BUN	CysC	Scr		
Franz RW[11]	2011	20(12/8)	21(17/4)	48–87	48–87	vascular surgery	0/1				oral 200 μg preoperative	4
Li B[24]	2011	34(34/0)	26(26/0)	69.4 ± 7.3	67.9 ± 6.8	CHD CAG/PCI	2/3	48 h		48 h	20 μg + NS 20 ml iv qd, 30 min preoperative, continue 3d postoperative	2
Li JZ[25]	2016	50(33/17)	53(34/19)	58.11 ± 9.7	56.84 ± 9.29	CHD PCI	1/6			24 h,72 h	10 μg + NS 100 ml iv drip qd, 1d preoperative, 3d postoperative	2
Liu H[26]	2013	30(16/14)	30(15/15)	55–74	55–75	DM PAG	0/0		24 h,48 h	24 h,48 h	10 μg + NS 10 ml iv qd, 3d preoperative	3
Liu Y[27]	2011	29(16/13)	29(19/10)	57.0 ± 7.5	59.4 ± 9.2	CHD CAG/PCI	4/13	48 h,72 h		48 h,72 h	10 μg + NS 50 ml iv pumping bid, 7d postoperative	2
Liu YY[28]	2011	82(51/31)	84(51/33)	60.8 ± 12.5	61.7 ± 12.8	CHD CAG/PCI	2/3			48 h	20 ng/kg/min, continue 6 h postoperative	2
Li XY[29]	2013	14(8/6)	15(11/4)	66 ± 12	66 ± 12	DM PAG or PTA	0/2		24 h,72 h	24 h,72 h	2 ng/kg/min, continue 6 h preoperative and 20 μg + NS 40 ml iv qd, the second day, 5d postoperative	3
Li YN[30]	2014	150(96/54)	150(54/96)	68.02 ± 7.03	68.49 ± 6.10	CHD CAG/PCI	4/13	72 h		72 h	10 μg + NS 100 ml iv drip qd, 0.5-1 h preoperative, 3d postoperative	3
Miao Y[31]	2013	154(120/34)	176(133/43)	79.08 ± 6.16	78.26 ± 6.61	CECT	14/39		24 h,48 h,72 h	24 h,48 h,72 h	0.4 μg/kg/day, 48 h preoperative, continue to 48 h postoperative	4
Su C[32]	2015	55(35/20)	51(33/18)	62.7 ± 10.8	63.5 ± 11.2	CHD PCI	2/7	72 h		72 h	10 μg + NS 100 ml iv drip qd, 1 day preoperative, 3d postoperative	2
Wang L[33]	2016	50(31/19)	50(32/18)	60.48 ± 4.51	60.5 ± 4.17	CHD CAG/PCI	2/8	72 h		72 h	20 μg + NS 40 ml iv drip qd, 30 min preoperative,0.5,1, 2d postoperative	2
Wang ZD[34]	2015	65(49/16)	63(48/15)	58.2 ± 10.8	59.1 ± 11.2	CHD CAG/PCI	7/17	24 h,48 h,72 h		24 h,48 h,72 h	10 μg + NS 20 ml iv qd preoperative, 7d postoperative	3
Xu R[35]	2012	30(13/17)	30(12/18)	60 ± 9	60 ± 11	CHD CAG/PCI	2/10			24 h,48 h	10 μg + NS 20 ml iv qd, preoperative, 7d postoperative	2
Yan HY[36]	2014	21(12/9)	19(11/8)	73.3 ± 6.3	75.1 ± 8.5	PAG	4/8	24 h,48 h,72 h	24 h,48 h,72 h	24 h,48 h,72 h	10 μg + NS 10 ml iv bid, 3d postoperative	2
Ye Y[37]	2006	28(25/3)	30(26/4)	70.28 ± 5.6	72.62 ± 9.15	PAG/CECT	6/14	48 h,72 h		48 h,72 h	20 μg + NS 20 ml iv, qd, 3d postoperative	2
Zhao HW[38]	2014	58(31/27)	58(39/19)	64 ± 9	65 ± 8	CHD DM PCI	2/9		72 h	72 h	10 μg + NS 100 ml iv drip qd, 1d preoperative, 4d postoperative	2
Zhong SG[39]	2014	50(38/12)	50(39/11)	63.9 ± 7.6	64.1 ± 8.0	CHD DM PCI	1/8	72 h		72 h	100 μg + NS 100 ml iv drip bid, 3d postoperative	2
Zhou DC[40]	2013	112(65)	103(61)	62.1 ± 11.1	63.2 ± 10.9	CHD CAG/PCI	8/19				10 μg + NS 10 ml iv bid, 1d postoperative, 7-10d postoperative	3
Zhu L[41]	2011	99(99/0)	98(98/0)	57 ± 15	57 ± 15	CHD CAG ± PCI	7/19	48 h	48 h	48 h	20 μg + NS 20 ml iv qd, 10d postoperative	2

